# Supplementary material for: Parkinson’s disease associated mutation E46K of α-synuclein triggers the formation of a distinct fibril structure
Source: Nat Commun. 2020 May 26;11:2643. doi: 10.1038/s41467-020-16386-3 (PMC7250837; doi:10.1038/s41467-020-16386-3)
Supplement: Supplementary file 1 — Supplementary Information [file 41467_2020_16386_MOESM1_ESM.pdf]

Supplementary Information

Parkinson’s disease associated mutation E46K of α-synuclein triggers the formation of a distinct fibril structure

Kun Zhao, Yaowang Li et al.

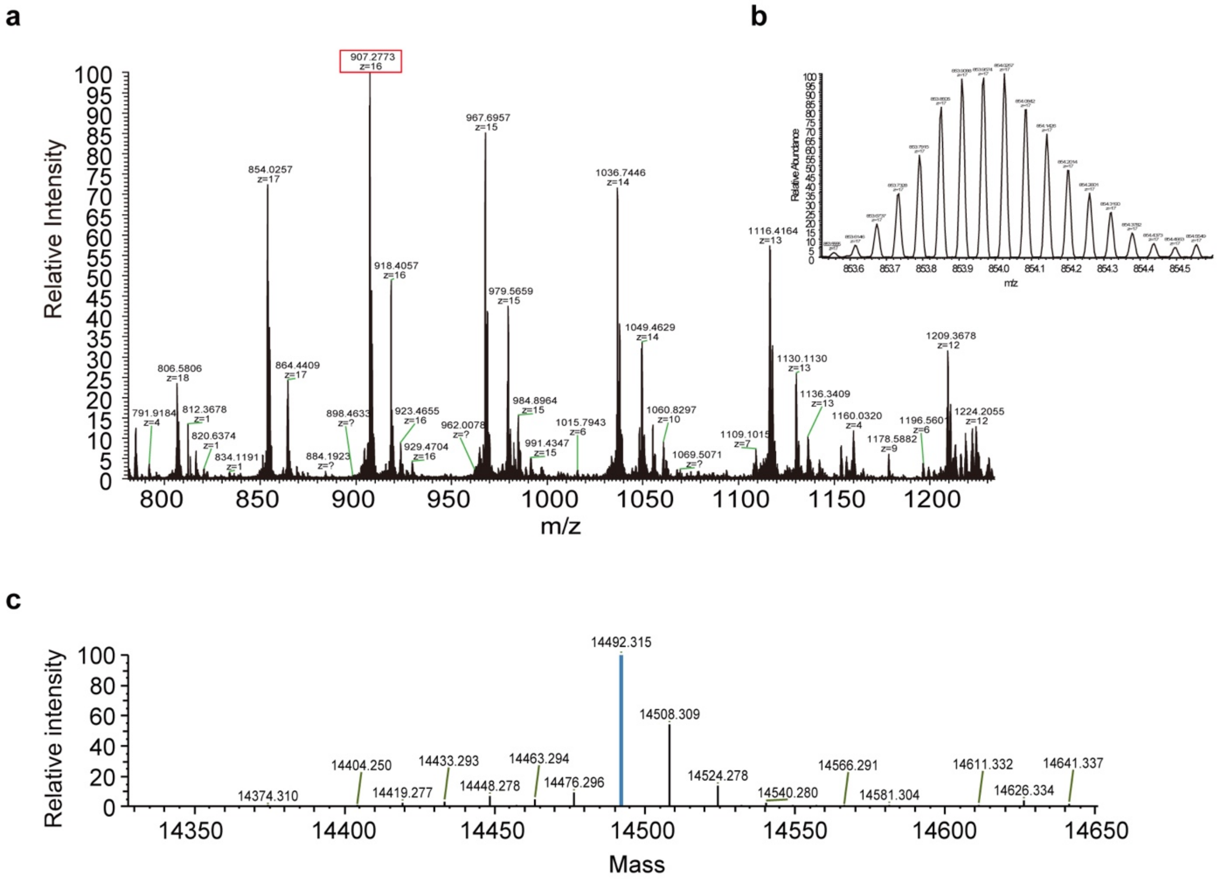

Supplementary Figure 1 MS validation of the recombinant Ac-E46K α-syn. **a** The different charge states of the Ac-E46K α-syn. **b** The isotopically resolved profile of the Ac-E46K α-syn. **c** The deconvoluted spectrum confirms that the protein is acetylated.

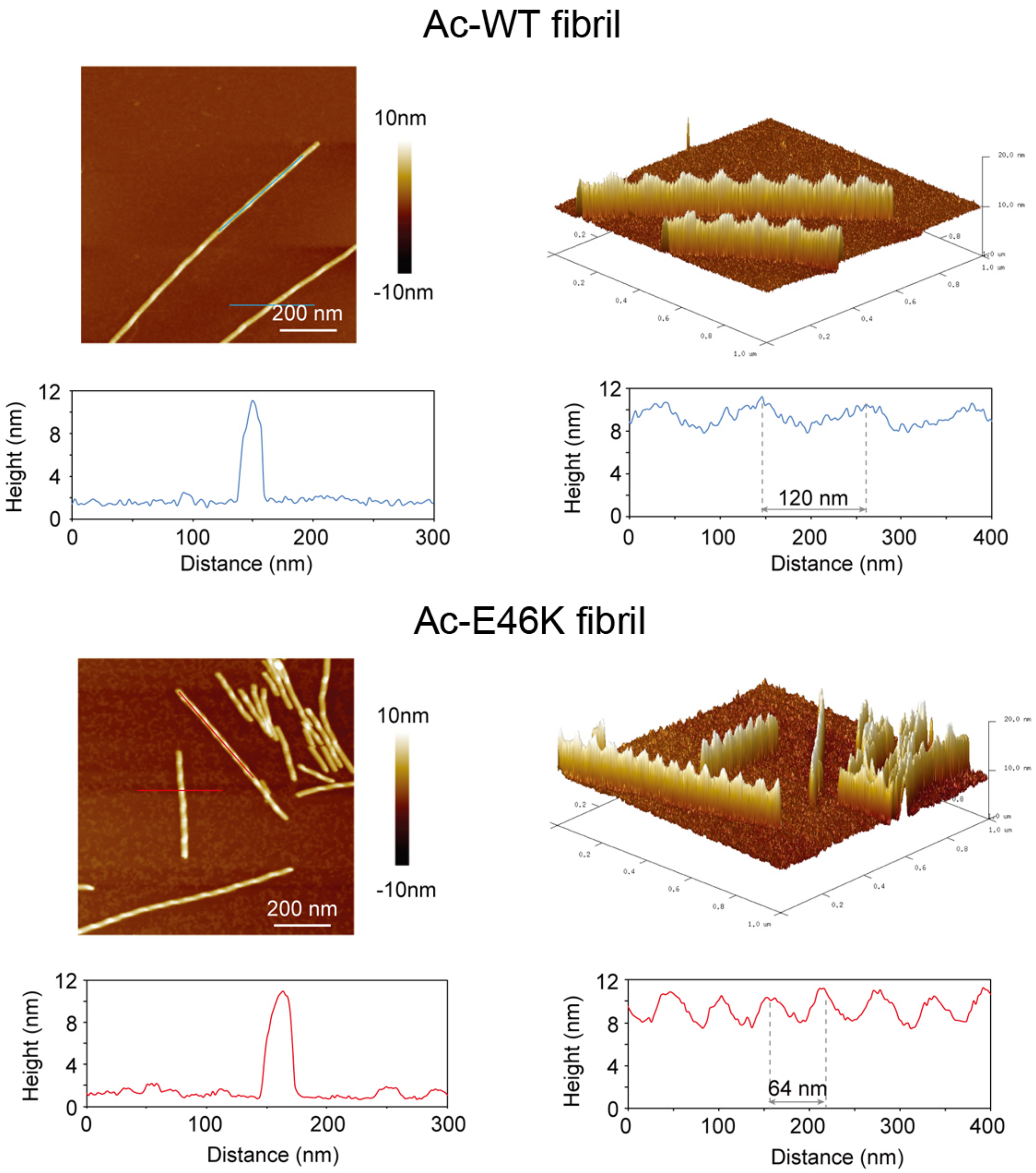

Supplementary Figure 2 AFM measurement of the Ac-WT and Ac-E46K α-syn fibrils. The fibrils were formed under the same condition: 50 mM Tris, 150 mM KCl, pH 7.5 at 37°C with agitation. AFM 2D and 3D images are shown. Analyses of the cross section and along the fibril are indicated with blue/red lines, respectively. The width and twist periodicity of the fibrils are measured.

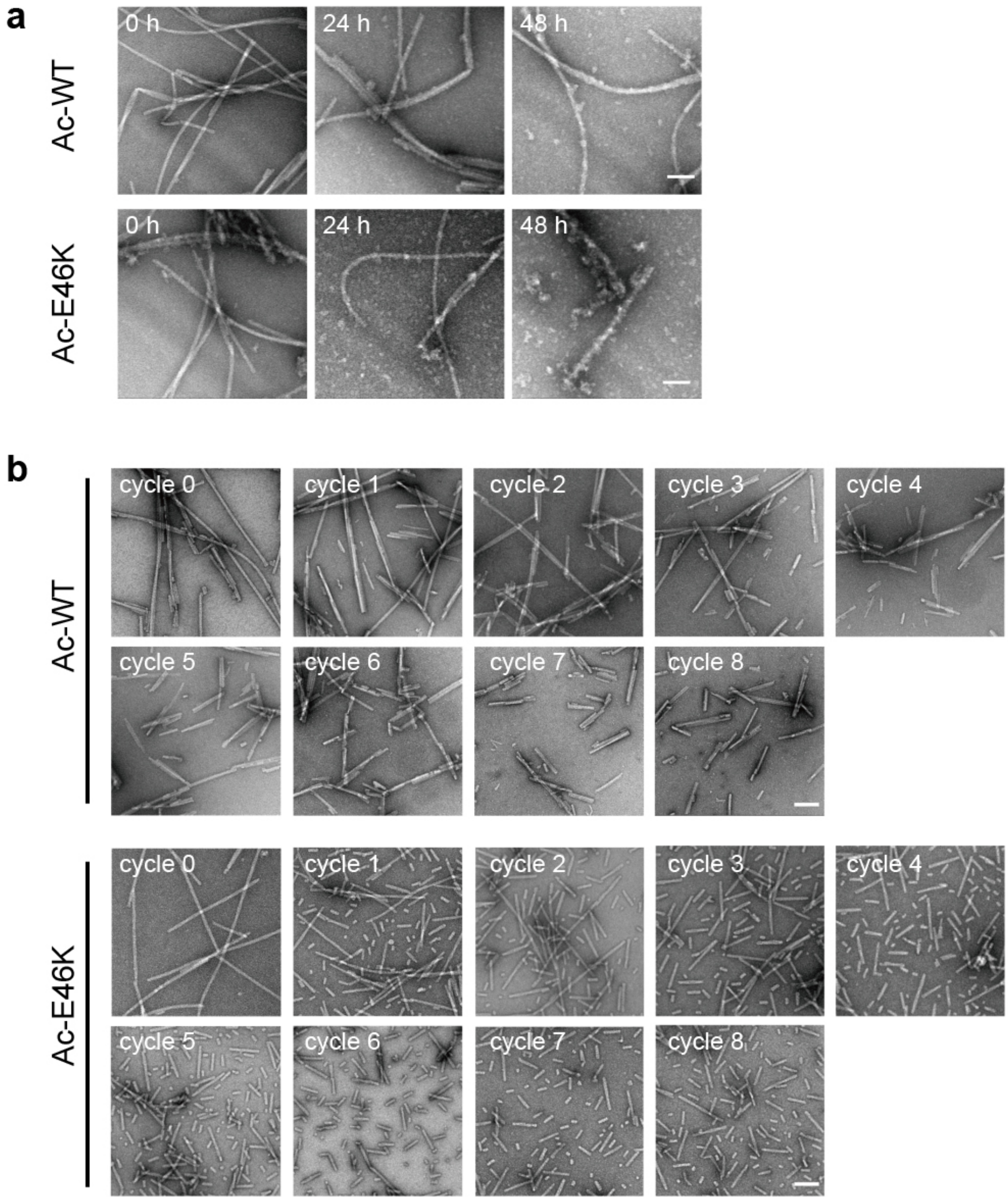

Supplementary Figure 3 Negative-staining TEM images of Ac-WT and Ac-E46K α-syn fibrils upon cold denaturing and freeze-thaw. Morphological changes of Ac-WT and Ac-E46K fibrils upon incubation at 0° for 24 h and 48 h (**a**) and upon 8 cycles of freeze-thaw (**b**). Scale bar: 100 nm. The experiment was repeated for >4 times independently with similar results.

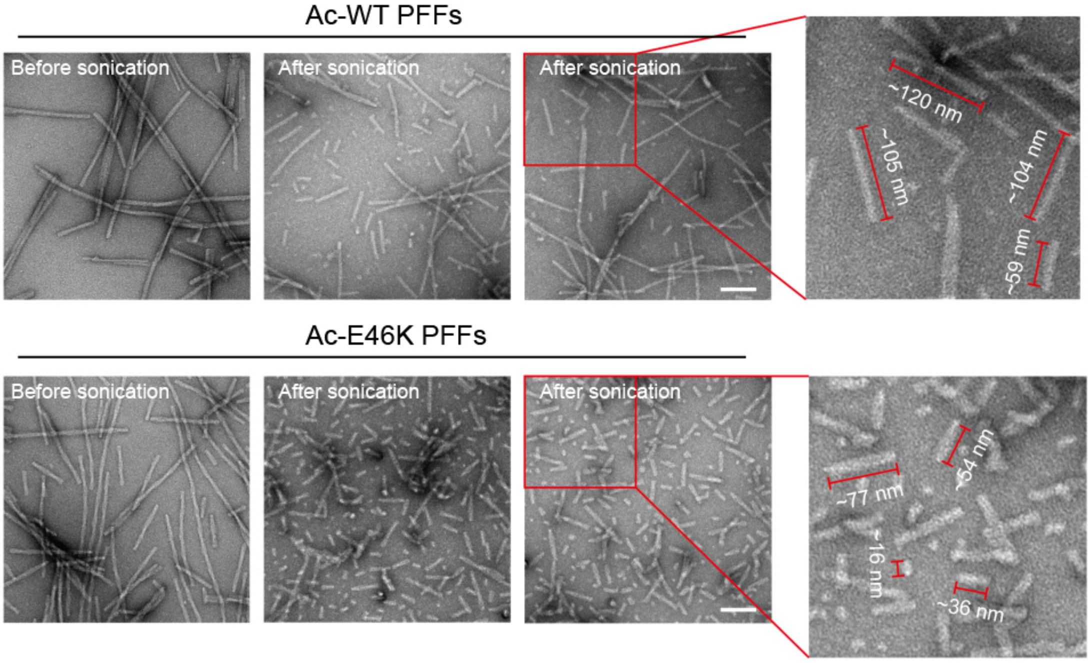

Supplementary Figure 4 Negative-staining TEM images of Ac-WT and Ac-E46K α-syn PFFs before and after sonication. Zoom-in views are shown on the right with fibril lengths marked. Scale bar: 100 nm. The experiment was repeated for >6 times independently with similar results.

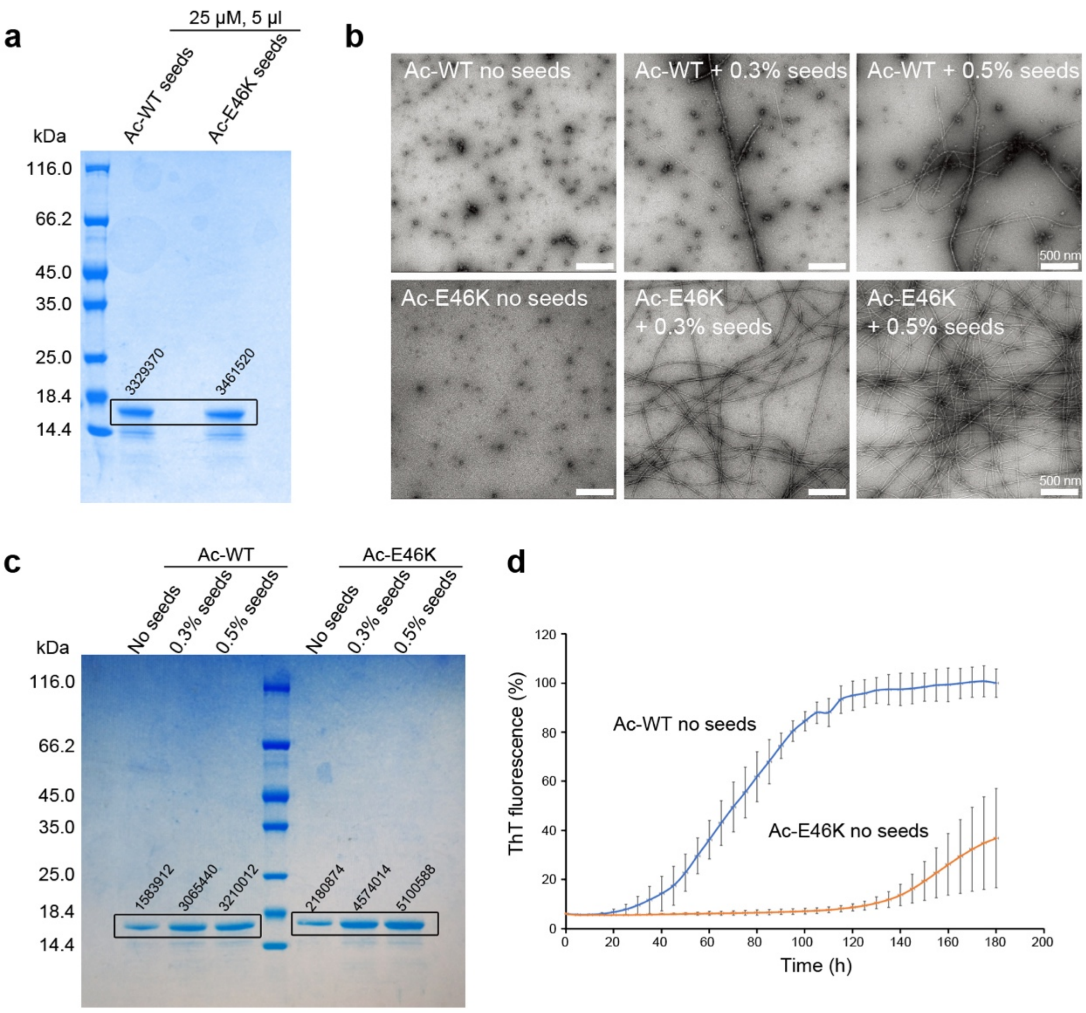

Supplementary Figure 5 Seeded or unseeded fibril formation of Ac-WT and Ac-E46K. **a**, SDS-PAGE of Ac-WT and Ac-E46K PFF seeds. Intensities of the major bands (framed) are indicated. **b**, Negative-staining TEM images of Ac-WT and Ac-E46K fibrils with/without PFF seeds. Images were taken after 80-h fibril growth. Scale bar: 500 nm. **c**, SDS-PAGE of the pellets in Ac-WT and Ac-E46K fibrillation samples. Samples were collected after 80-h fibril growth. Same volumes of loading buffer were applied to resuspend the pellets. Same volumes of each sample were loaded on the gel. Intensities of the major bands (framed) are indicated. **d**, ThT kinetic assay of Ac-WT and Ac-E46K without PFF seeds. Data shown are mean ± s.d., n=3 biologically independent samples. Each experiment was repeated for >3 times independently with similar results. Source data are provided as a Source Data file.

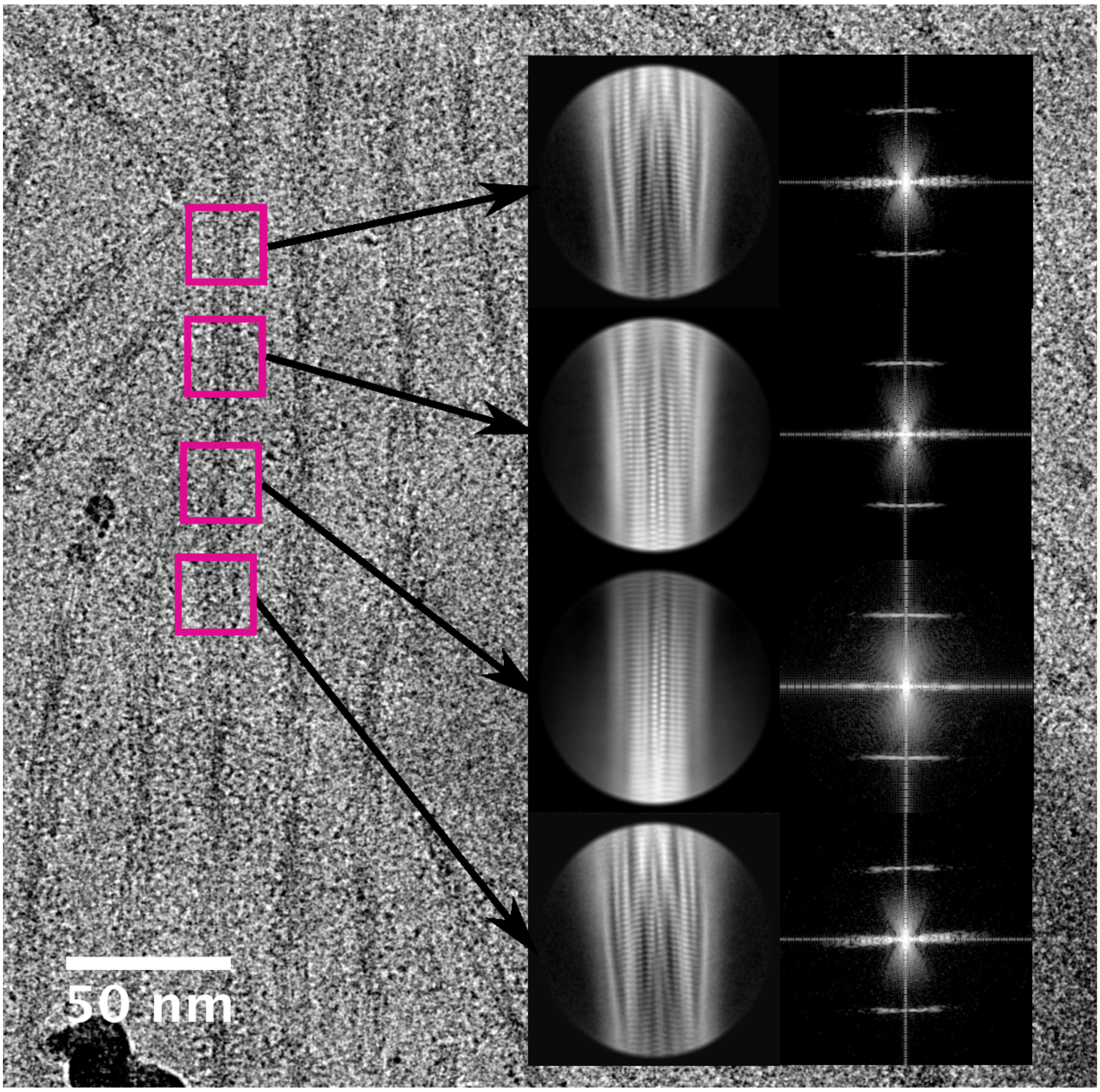

Supplementary Figure 6 2D classification of the Ac-E46K fibril. A cryo-EM micrograph of the Ac-E46K fibril is shown. The zoom-in views show the 2D class averages and power spectra of the indicated fibril segments. 13,064 filaments were manually picked from 754 independent micrographs.

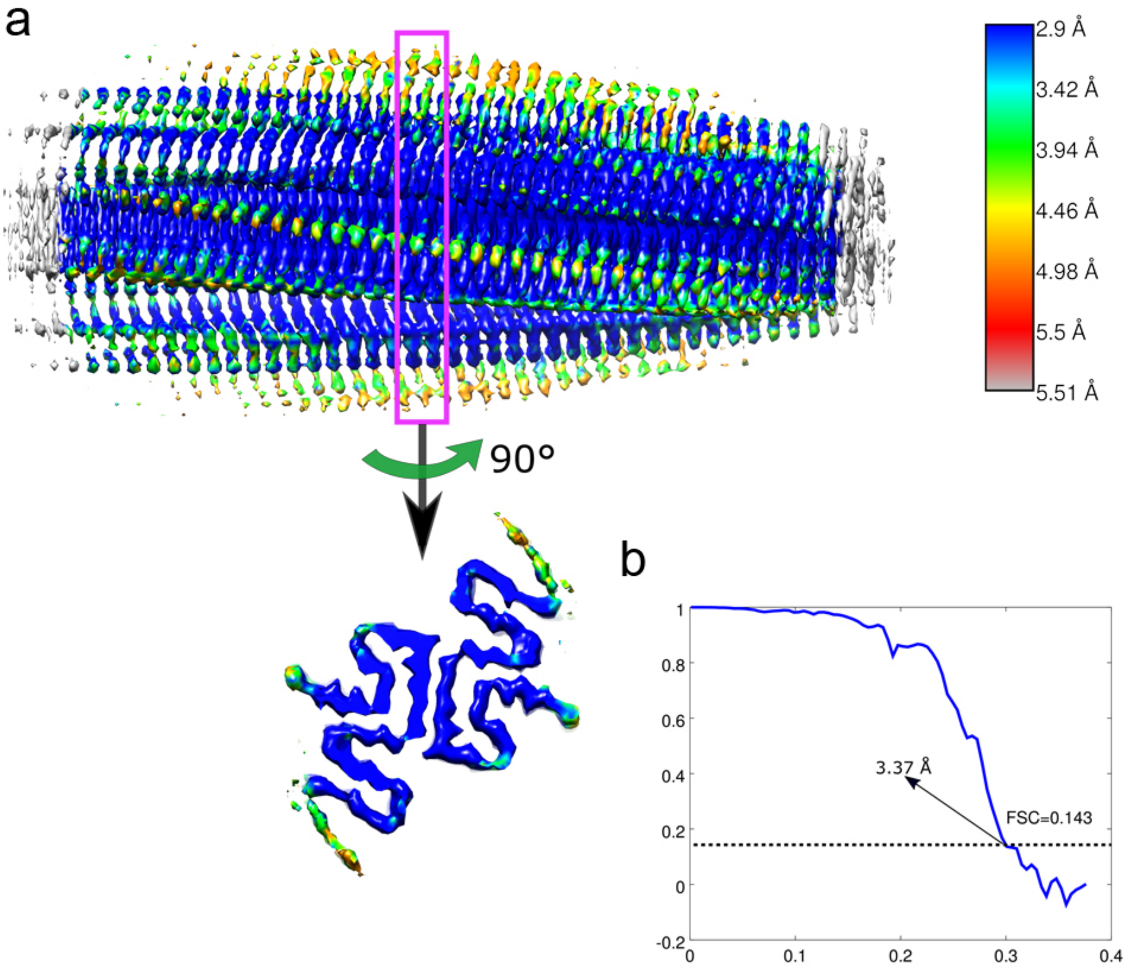

Supplementary Figure 7 Resolution estimation of the cryo-EM structure of Ac-E46K α-syn fibril. **a** Local resolution estimation. EM reconstruction maps are colored based on the local resolutions. The color scale indicates the resolution ranging from 2.90 Å to 5.51 Å. **b** Gold-standard Fourier shell correlation curve of the Ac-E46K α-syn fibril. The overall resolution is 3.37 Å.

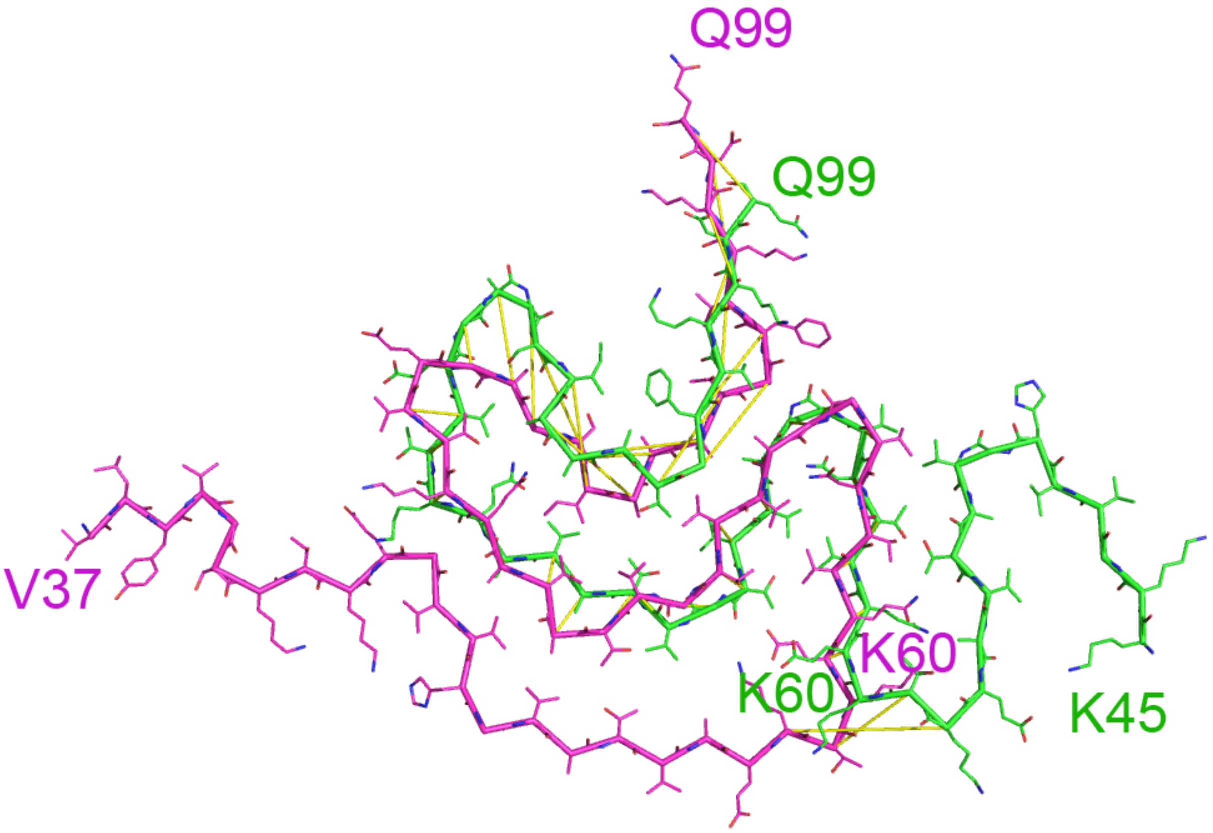

Supplementary Figure 8 Alignment of the Ac-E46K (green) and Ac-WT (magenta) α-syn subunit structures. The PDB ID of the Ac-WT fibril structure is 6A6B. The FC-N regions of the two structures diverge at K60. The FC-C regions of the two structures adopt a similar topology, while their structural similarity is low with an r.m.s.d. of Cα atoms of 5.558 Å. Yellow lines highlight the discrepancy between the same residues of the two structures.

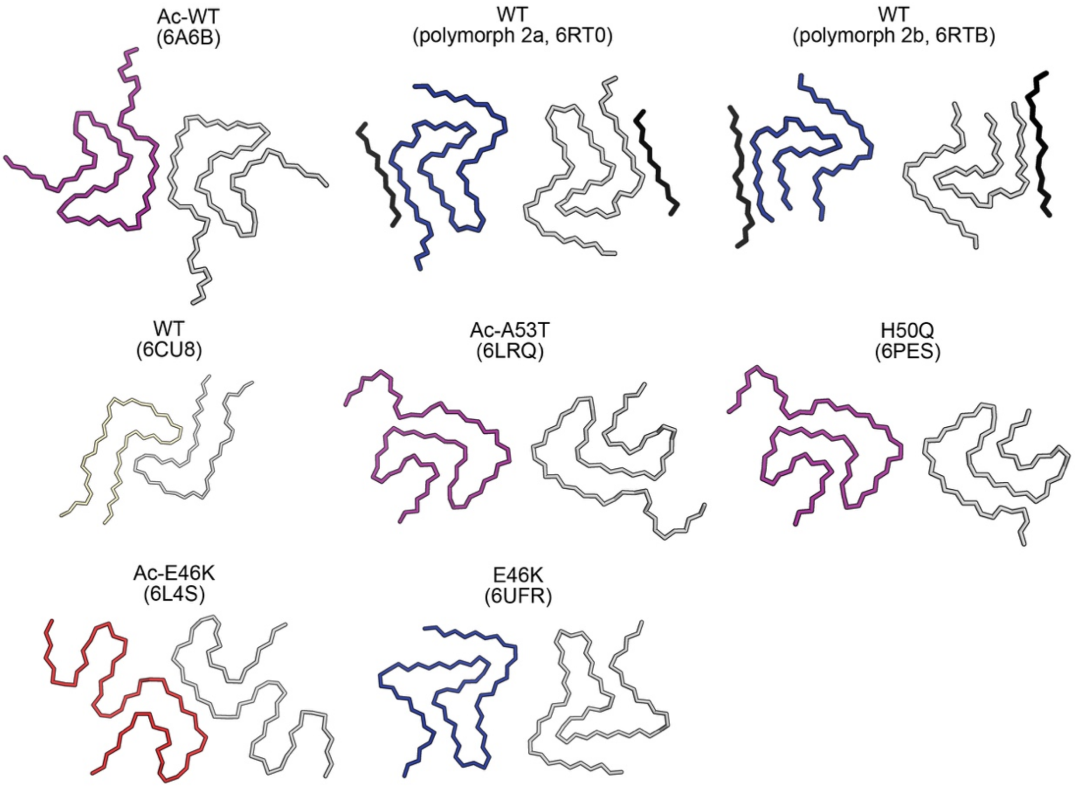

Supplementary Figure 9 Polymorphic fibril structures formed by WT and mutant α-syn. One layer composed of two α-syn molecules of each fibril structure is shown. Similar folds of α-syn monomer are colored the same. PDB IDs of the structures are provided in parentheses.

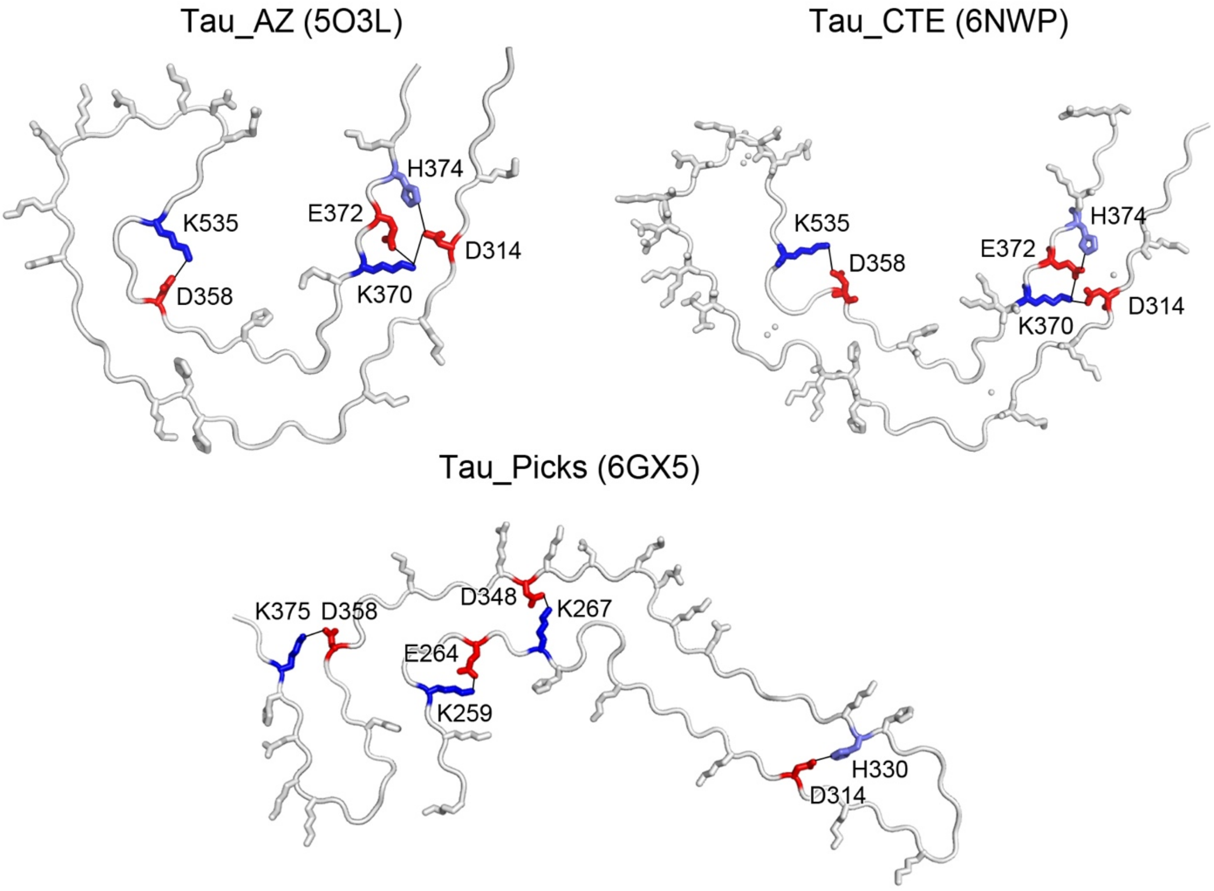

Supplementary Figure 10 Electrostatic interactions in tau fibril polymorphs. One layer of different polymorphic structures of tau fibrils. Single tau subunits are shown since there is no inter-molecular electrostatic interaction in the tau fibrils reported so far. Electrostatic interactions are highlighted with K colored in blue, H in light blue and E/D in red. PDB IDs of the structures are provided in parentheses.

Supplementary Table 1 primer sequences used in this study.

Primers for α-syn WT	
α-syn-F	TTTTTCATATGGATGTATTCATGAAAGG
α-syn-R	TTTTTTCTCGAGTTAGGCTTCAGGTTCGTAGTC
Primers for E46K point mutation	
E46K-F	CAAGAAAGGAGTGGTGCATGGTGTGG
E46K-R	CTCCTTTCTTGGTTTTGGAGCCTAC
